# KNOWLEDGE GAPS REGARDING NALOXONE USE AMONG OLDER ADULTS IN HIGH OPIOID PRESCRIBING REGIONS IN SOUTHERN USA

**DOI:** 10.1093/geroni/igae098.4012

**Published:** 2024-12-31

**Authors:** Gohar Azhar, Naomi Armstrong, Lauren Camp, Karen Coker, Patricia E Savary, Shakshi Sharma, Regina Gibson, Jeanne Y Wei

**Affiliations:** University of Arkansas for Medical Sciences, Little Rock, Arkansas, United States; University of Arkansas for Medical Sciences, Little Rock, Arkansas, United States; University of Arkansas for Medical Sciences, Little Rock, Arkansas, United States; University of Arkansas for Medical Sciences, Little Rock, Arkansas, United States; University of Arkansas for Medical Sciences, Little Rock, Arkansas, United States; University of Arkansas for Medical Sciences, LITTLE ROCK, Arkansas, United States; University of Arkansas for Medical Sciences, Little Rock, Arkansas, United States; University of Arkansas for Medical Sciences, Little Rock, Arkansas, United States

## Abstract

Arkansas has one of the highest opioid prescribing rates in the country. Nevertheless, there is a dearth of knowledge regarding opioid use and naloxone among older adults. We surveyed community dwelling older adults about opioid risks and naloxone use in rural Arkansas. Total number of respondents was n=461, and the age ranged from 50-59 (22%), 60-69 (34%), 70-79 (30%), or 80+ (14%) with 77% females and 52% black or African Americans. The percentage correct answers received from survey respondents decreased as age increased. Older adults underestimated their risk for opioid overdose (older vs younger; 35% vs 16%, P=0.020). In addition, more older adults believed that only heroin or fentanyl users were likely to overdose on opioids (older vs younger; 85%,vs 68% P=0.029). The use of naloxone as an overdose antidote was much less recognized by older adults (older vs younger; 66% vs 87%, P=0.016), as well as the requirement to administer as second dose of naloxone if the initial dose was unsuccessful (older vs younger; 40% vs 63%, P=0.033). Most older adults were unaware that naloxone could be obtained from the pharmacy without a prescription (older vs younger; 47% vs 71%, P=0.018). In summary, knowledge gaps regarding opioids and naloxone use were significantly higher in older adults. Since older adults are frequently prescribed opioids for pain, it is imperative that greater educational efforts are directed towards ameliorating the knowledge deficits about opioids and correct use of naloxone to prevent adverse effects and deaths due to opioid overdose.

